# Addressing Hypertension Outcomes Using Telehealth and Population Health Managers: Adaptations and Implementation Considerations

**DOI:** 10.1007/s11906-022-01193-6

**Published:** 2022-05-10

**Authors:** Connor Drake, Allison A. Lewinski, Abigail Rader, Julie Schexnayder, Hayden B. Bosworth, Karen M. Goldstein, Jennifer Gierisch, Courtney White-Clark, Felicia McCant, Leah L. Zullig

**Affiliations:** 1 Department of Population Health Sciences, Duke University School of Medicine, 215 Morris Street, Durham, NC 27701 USA; 2 Durham Center of Innovation to Accelerate Discovery and Practice Transformation, Durham Veterans Affairs Health Care System, Durham, NC USA; 3 Division of General Internal Medicine, Department of Medicine, Duke University School of Medicine, Durham, NC USA; 4 Department of Psychiatry and Behavioral Sciences, Duke University School of Medicine, Durham, NC USA; 5 School of Nursing, Duke University, Durham, NC USA

**Keywords:** Hypertension, Population health management, Implementation science, Multidisciplinary care team, Electronic health record, Telemedicine

## Abstract

**Purpose of Review:**

There is a growing evidence base describing population health approaches to improve blood pressure control. We reviewed emerging trends in hypertension population health management and present implementation considerations from an intervention called Team-supported, Electronic health record-leveraged, Active Management (TEAM). By doing so, we highlight the role of population health managers, practitioners who use population level data and to proactively engage at-risk patients, in improving blood pressure control.

**Recent Findings:**

Within a population health paradigm, we discuss telehealth-delivered approaches to equitably improve hypertension care delivery. Additionally, we explore implementation considerations and complementary features of team-based, telehealth-delivered, population health management. By leveraging the unique role and expertise of a population health manager as core member of team-based telehealth, health systems can implement a cost-effective and scalable intervention that addresses multi-level barriers to hypertension care delivery.

**Summary:**

We describe the literature of telehealth-based population health management for patients with hypertension. Using the TEAM intervention as a case study, we then present implementation considerations and intervention adaptations to integrate a population health manager within the health care team and effectively manage hypertension for a defined patient population. We emphasize practical considerations to inform implementation, scaling, and sustainability. We highlight future research directions to advance the field and support translational efforts in diverse clinical and community contexts.

## Introduction

Hypertension (HTN) affects approximately 116 million adults in the USA [1]. It is one of the most prevalent contributors to cardiovascular disease, resulting in approximately 659,000 deaths in the USA each year [2, 3]. While HTN alone is associated with cardiovascular disease and “all cause” mortality, its high co-occurrence with obesity, dyslipidemia, and diabetes makes HTN a persistent obstacle towards improved outcomes and equity and a significant driver of costs. When adjusting for age, the burden of HTN and related chronic conditions falls disproportionately on underserved communities [4]. As such, improving HTN outcomes among underserved communities is a priority area of focus for population health management efforts. Population health management is defined by its proactive focus on the health needs of a defined population or community, in contrast to exclusively reacting to individual level patient medical needs when they arise [5, 6]. Within a population health paradigm, HTN management leverages technologies to identify and engage at-risk populations, thereby increasing reach and the potential for impact particularly among underserved communities.

There are multiple evidence-based strategies to manage HTN, including lifestyle modifications and medications. When HTN control is achieved, stroke is reduced 35–40%, myocardial infarction 20–25%, and heart failure over 50% [7, 8]. However, uptake of lifestyle modifications and adherence to medications is suboptimal, so HTN control is often not achieved. In an analysis of 2015–2018 data, only 26.1% of adults with diagnosed HTN and prescribed medication achieved HTN control [9]. Patients’ long-term non-adherence rate to provider’s pharmacotherapy recommendations range from 50–70% [10]. Patient-level barriers associated with this lack of treatment and self-management include suboptimal adherence to medication or behavioral interventions and lack of knowledge and self-efficacy specific to managing HTN [11, 12]. Provider-level barriers to evidence-based HTN management include insufficient time to spend with patients and clinical inertia which can result in failure to intensify treatments [13–15]. In fact, two-thirds of all suboptimal HTN treatment in the USA originates from inadequate provider treatment [16]. Provider challenges related to the complexity of polypharmacy is associated with suboptimal treatment adherence, which in turn leads to poor HTN control [17]. System level barriers such as a lack of care coordination, decision support, and access to BP monitoring/follow-up exacerbate existing challenges to HTN treatment [18]. The cumulative effect of barriers at the patient, provider, and system level is suboptimal HTN treatment. To be effective, population health management initiatives must recognize and work within and across each of these levels to achieve optimal blood pressure control targets.

In practice, population health management often refers to a range of health care services and analytics that support a coordinated and proactive approach to support improved outcomes for a community. In other words, the goal of population health management is to improve the health and well-being of a specific group of people or community [19••]. To this end, population health management uses multilevel approaches that require careful attention to the broader context of patient situations (e.g., low-income communities, limited transportation options, nutritious food availability, beliefs and norms, rurality) to better understand context-specific drivers of outcomes. By taking these multilevel factors into account, models of care can be more tailored and responsive.

We summarize emerging evidence to support HTN population health management as an effective responsive to the multi-level drivers of inequitable outcomes. First, we highlight several distinct yet complementary components of emerging best practices for designing HTN population health management initiatives: (1) population health management and the role of a population health manager; (2) team-based care; (3) telehealth modalities; and (4) the electronic health record (EHR). Second, we describe how the chronic care model can be used to guide the design of multi-level interventions. Third, we use the TEAM intervention as a case study to present implementation considerations and intervention adaptations to effectively manage HTN for a defined patient population. In describing the implementation processes, we emphasize practical considerations associated with intervention design, adaptation, scaling, and sustainability in real-world contexts. Finally, we provide perspective on future research directions and opportunities to support the translation of TEAM and HTN population health approaches like it into practice.

## Components of Approaches to HTN Population Health Management

1. Population Health Management and the Role of a Population Health Manager

### Population Health Management

Serxner et al. described the core components of population health management including attention to the full continuum of care, data sharing, and multidisciplinary care coordination [20]. Similarly, Matthews et al. focused on the shift of the role of health care systems from sporadic and episodic encounters to serve individuals in need of immediate care towards accountability for the health and well-being of an entire patient population [21]. When treatment interventions are organized and delivered through a population health management paradigm, interventions have the potential to lessen health disparities by proactively focusing on both unmet clinical and health-related social needs. Examples of population health management interventions to promote health equity include implementation of a system-wide screening intervention to decrease disparities in colorectal cancer screening rates [22], a care-management intervention bridging the transition from in-patient to out-patient mental health care to reduce readmissions of at-risk patients [23], and community partnerships and coordination to promote treatment adherence [24, 25•]. Additionally, interventions based on population health management are effective in achieving BP control for patients with uncontrolled HTN. For example, in a propensity score-matched cohort study of patients that received multi-disciplinary team case management to address HTN disparities in Baltimore, patients that completed all three sessions (39%) experienced reductions in systolic (9 mmHg) and diastolic blood pressure (4 mmHg) greater than matched controls with no disparities in these reductions among White and Black patients [26•].

By taking a population health approach, we consider how inequities and social determinants of health shape why certain populations have differential health outcomes [27]. As population health management continues to gain prominence in the context of value-based care [28], it is critical to design HTN interventions and support translational efforts to ensure use in clinical practice. To translate evidence-based population health management interventions into practice, there are considerations that may impact the roles, responsibilities, composition, and staffing priorities of the care team and health system.

### Population Health Manager

One emerging role that is central to population health management is that of the population health manager. A population health manager is a member of the health care team who uses population-level health data to identify unmet needs, intervene, and improve inequities and clinical outcomes among a defined community, usually within a shared geographic area. When embedded within the care team, the population health manager provides culturally appropriate counseling and gives support to patients related to their health self-management, navigating community resources or social services, and promoting treatment adherence [29–31]. Furthermore, a population health manager considers patient-level factors (e.g., self-efficacy, medical needs, social needs), clinic-level factors (e.g., specialty providers, community-based care, wrap-around service availability), and system-level factors (e.g., physical environment, eligibility and access to health and social services, and locally available resources), when developing health strategies [32, 33]. Many times, nurses fill the role of population health managers as the role, purpose, and function of the population health manager complement the nurse’s training and approach to health and wellness.

Within the context of HTN, a population health manager would consider the patients’ cultural preferences and environment to tailor strategies for promoting self-management and medication and treatment adherence for HTN risk reduction. The reorientation of the health care workforce towards population health management, including the adoption of population health managers into care teams, represents an important opportunity to improve population health HTN outcomes and complement providers’ clinical management activities.

2. Team-Based Care

Team-based care is an essential component of a population health approach for HTN management. Team-based care models include personnel from both clinical specialties (e.g., social work, medicine, nursing) and non-clinical specialties (e.g., community health worker, data science, informatics) and are an evidence-based approach to improve health outcomes and quality of care, especially for those with chronic conditions. Effective team-based care models focus on disease management, provider coordination, performance measurement, the process of care delivery, and engaging the patient in decision making to promote self-management [34]. A team-based approach to HTN care is ideal but can be challenging in practice; for example, a patient’s primary care provider may not be able to easily view records and information generated by other providers. As a result, medication non-adherence or non-optimal prescribing patterns can result from lack of continuity of care due to barriers to information sharing [15, 35]. A change in health behavior due to team-based care is particularly relevant to a condition such as HTN in which sustained treatment adherence and self-management are critical.

HTN team-based care interventions are associated with improved BP control and lower systolic and diastolic BP (especially when pharmacists and nurses are involved) and predict effectiveness of drugs [36••, 37•]. Specifically, a 2009 meta-analysis by Carter at el. found that HTN interventions that emphasized integrating complementary roles and clinical specialties had large effect sizes on systolic blood pressure (SBP). For example, effective team-based care strategies included pharmacist treatment recommendations (− 9.30 mm Hg), nurse led interventions (− 4.80 mm Hg), and use of a treatment algorithm enabled through clinical informaticist support (− 4.00 mm Hg) [36••]. Team-based care not only has the potential to improve HTN outcomes, but also represents an extremely cost-effective approach to HTN management [38•, 39]. A 2015 economic systematic review of team-based care interventions to control blood pressure found that they almost all were cost-effective at the most conservative threshold of $50,000 per quality-adjusted life-year (QALY) [40]. In addition, studies demonstrate that team-based care can increase patient satisfaction [40]. There is also an emerging evidence base that team-based care may be a mechanism for improving health equity. An analysis of 12 systematic reviews of the literature describing promising patterns and designs of interventions to reduce health disparities identified multidisciplinary team-based care interventions that were culturally tailored as a promising strategy [41].

3. Leveraging Telehealth Modalities

Effective population health management should consider strategic integration of existing technologies and digital platforms including telehealth to engage patients [42•]. Telehealth is when a healthcare system exchanges medical information through electronic communication platforms to address a patient’s health [43, 44]. Telehealth enabled communication can occur between clinicians, clinician and patient, and patient and mobile health technology to perform a variety of services including counseling, medication management, education, and monitoring. Telehealth occurs via two-way synchronous video or audio-only (e.g., by telephone) communication to complement or substitute in-person encounters as an efficient and convenient, accessible mechanism for chronic care delivery. Notably, the COVID-19 global pandemic and associated changes to telehealth reimbursement and regulatory policies have accelerated adoption of telehealth infrastructure across medical specialty service lines, a trend that continued after in-person visit volume returned to pre-pandemic levels [45].

There is an emerging evidence base describing effective telehealth approaches for patients with HTN and related chronic conditions. Specifically, telehealth technologies represent an important opportunity to improve HTN population health by addressing the multi-level drivers of blood pressure control. For example, in a 4-year randomized control trial, a nurse-led behavioral intervention administered via telephonic encounters showed an increase in HTN control by 13% when compared to usual care [46]. Another recent study showed that when used in conjunction with electronic health databases, telehealth interventions can improve quality of life, disease stability, and treatment compliance in elderly patients with co-morbid diseases [47]. Telehealth interventions are a promising mechanism for modifying patient non-adherence behaviors [48, 49]. A systematic review of telehealth application included a broad range of clinical applications and showed that telehealth interventions as adjuncts to routine care produces positive outcomes and that the most pronounced benefits were for chronic conditions like HTN [45]. For example, telephone-based interventions improve treatment adherence and blood-pressure control in individuals with HTN [50•]. Telehealth interventions also show promise for promoting health equity. When compared to usual care, nurse implemented telemedicine that combined behavioral and medical interventions improved mean systolic blood pressure, especially in African Americans [51]. Telephone-administered behavioral interventions have also shown success in HTN control and promise in Medicaid patients [52, 53•, 54, 55, 56•, 57, 58]. For example, in one 2011 study, implementing a telehealth HTN behavioral intervention resulted in treatment adherence increase from 55 to 77% in Medicaid patients [53•]. One study implementing a telephone administered behavioral intervention at three primary care clinics versus nine usual care sites showed success in larger-scale implementation; however, there is still a gap between discovery and delivery. Despite evidence that these interventions can be effective in controlled research conditions, there is a need to better understand (i) what is necessary to introduce and sustain a novel intervention into routine clinical care and (ii) what aspects of the intervention are critical to preserve to produce the same results in “real-world” clinical and community settings [52]. As telehealth becomes a more common treatment intervention, health system-wide data for tailored scaling across a defined population is a critical need and implementation consideration [59].

4. The Role of the EHR

Effective and efficient HTN care models often feature the use of EHR technologies to identify patients, coordinate activities, and engage through behavioral interventions in a timely manner. In fact, emerging technologies related to telemonitoring’s capture and integration within the EHR may represent an important opportunity for the field to augment and synergize with team-based care [60••]. Thus, there is an important role for system-level investments in clinical information systems and technology infrastructure to facilitate HTN team-based care and population health management.

Population health management interventions that leverage the EHR can improve population health by identifying high risk patients. EHRs include physical assessments and medical histories of individual patients to assess cardiovascular risk and rising risk [61]. EHRs can be used to evaluate interventions and predict risk, especially when integrated with other sources including data on social determinants [56•, 62]. For example, data obtained from EHRs can be used to predict development of HTN and coronary heart disease, or to track associations between hospitalizations and patient characteristics related to heart-failure [63–65]. Additionally, EHRs can be used to assess health disparities [66] and inform the development of interventions to improve both health equity and outcomes.

The use of EHRs to inform interventions has implications for the design of population health surveillance efforts. New York City created a system to track HTN prevalence using aggregate EHR data from a network of outpatient practices. This example represents an opportunity to leverage EHR infrastructures at the population level and use the EHR to go beyond syndromic surveillance and quality improvement at the individual and practice levels [67, 68]. Similarly, when a health maintenance organization system implemented a population-based HTN management program, they were able to achieve above 80% blood pressure control across the population represented in the hypertension registry [69•]. A key facet of the program was a comprehensive, EHR-based, HTN registry to enable customizable queries that informed prioritization of patient subgroups (e.g., individuals with poorly controlled hypertension, underserved communities) that would benefit from treatment intensification. This risk stratification is consistent with a review of HTN interventions that found the most effective approaches included population (e.g., clinic or panel-level review) rather than an exclusive focus on patient-level intervention [70]. Using EHRs to enable a HTN population program deployed in concert with a team-based telehealth approach is synergistic and facilitates response to patient, provider, and system level barriers to HTN management.

## Applying the Chronic Care Model to Design HTN Population Health Management Approaches

A HTN intervention within a population health paradigm should include an inclusive approach which incorporates chronic care model (CCM) concepts, including self-management support, care coordination, enhanced clinical information systems (e.g., clinical registries and telehealth), and decision support [71]. Therefore, data-driven (e.g., EHR), team-coordinated, telehealth technologies and intervention strategies facilitated by a population health manager represent complementary approaches to advance HTN population health management. We conducted a narrative review to describe the evidence base of these synergistic strategies that fit seamlessly within a population health management care paradigm. Table 1 summarizes particularly relevant studies the describe the role of a population health manager and the use of team-based care, the EHR, and telehealth to better manage HTN.

**Table 1:** Selected studies evaluating recommended components of HTN population health management

**Author (year)**	**Study design**	**Setting**	**Intervention description**	**Findings**
*Population health manager interventions*				
Hussain [26•]	Quasi-experimental, study	Six primary care practices in the Baltimore metropolitan region. 4/6 clinics were in medically underserved areas/	Care management intervention designed to reduce disparities between Black and White patients in routine clinical environments. Intervention consisted of (i) EHR based identification and proactive outreach; (ii) PHM/care manager with expertise in lifestyle counseling; and (iii) other members of care team (e.g., pharmacists). Intervention delivered via telephonic outreach over 3 sessions, 4 weeks apart (120 min of engagement)	Patients that completed the intervention (*n* = 229) experienced a 9 mm Hg SBP greater improvement compared to a matched cohort of non-participants (*n* = 330) and a 5 mm Hg greater improvement over partial completers (*n* = 332). The following implementation challenges were noted: • Reach was poor among the target population, but participants were representation of the target population • Only 40% of patients completed all 3 sessions
Milani [42•]	Quasi-experimental study	Integrated health system located in southeastern Louisiana	Patients were separated into two groups: those provided a home-based digital-medicine blood pressure program and a control (usual care). Those provided the digital program were given questionnaires, submitted at least one blood pressure reading per week, and were given lifestyle and medication management techniques from pharmacists and health coaches/PHMs	71% of digital program participants (*n* = 156) achieved blood pressure control compared to 31% of the control group, with a decrease of 14/5 mm Hg compared to 4/2 mm Hg. Analysis revealed: • A digital health intervention is feasible in improving BP control and patient activation • Approaches centering on a patient’s health capability significantly impact uptake of BP control
Halladay [25•]	Study protocol for cohort study	Six primary care practices in Eastern North Carolina	The practice intervention: (1) one member of the practice joined the “design team,” (2) stakeholders were invited to quarterly dinner meetings, (3) a practice “facilitator” engaged staff in the design, (4) a staff member assessed HTN control and collaborative partnerships, (5) practices delivered medication algorithm, (6) practices asked to use visit planner and decision support Patient intervention: (1) patients instructed for home BP, (2) patients called by coach for intervention feedback	Results from patient and practitioner interviews centered around a community-based participatory research approach in conjunction with qualitative analysis created the components of the intervention
*Team-based care interventions*				
Carter [36••]	Systematic review	Not applicable	The review focused on interventions to improve blood pressure control that involved nurses or pharmacists as part of a team-based approach. 37 articles met inclusion criteria	The following intervention components were associated with significant improvements in blood pressure control: education about medications (reduction in mean blood pressure of − 8.75/ − 3.60 mm Hg); pharmacist treatment recommendations (− 9.30 mm HG of systolic blood pressure); intervention by nurses (− 4.80 mm HG of systolic blood pressure); treatment algorithms (− 4.00 mm HG of systolic blood pressure)
Jacob [38•]	Systematic review	Not applicable	The review focused on costs related to interventions (and effectiveness), cost averted, and benefit-to-cost. 31 articles met inclusion criteria	The cost of intervention in relation to reduction in BP was converted to cost per quality-adjusted life-year (QALY). Team-based care is cost-effective (based on 10 studies assessing $/QALY)
Proia [37•]	Systematic review	Not applicable	The review focused on the effectiveness of team-based care interventions on improving BP and BP outcomes. 80 studies qualified for inclusion, a combination of a previous systematic review and a community guide update	Team-based care effectively improves BP outcomes. Information added by the community guide update included increases in percentage points for controlled BP (median proportion increase = 12), decreased systolic BP (median decrease = 5.4), and decreased diastolic BP (median decrease = 1.8 mmHg)
*Telehealth interventions*				
Bosworth [56•]	Randomized controlled trial	Two university-affiliated primary care clinics located in Durham, North Carolina	Enrolment sites were stratified, and 636 hypertensive patients were randomly assigned to usual care or combinations of 2 self-management interventions: a behavioral intervention (bimonthly telephone call by nurse addressing BP control behaviors), a group with BP home monitoring 3 × a week, and a combination	The behavioral care group had a proportion of 4.3% patients with BP control, the home BP monitoring had 7.6%, and the combination group had 11.0% relative to the controls. The combined group with both the behavioral telehealth intervention and home blood monitoring most effectively improved BP control and systolic/diastolic BP
Bosworth [53•]	Quality improvement	14 community-based networks that make up the statewide Medicaid patients from the statewide Community Care of North Carolina (CCNC) program	Patients received individually tailored calls that focused on lifestyle and medication adherence (tailored behavior self-management intervention)	Medication possession increased by 22 percentage points after the program (*n* = 558). The telephone interactions allowed the program to be specifically tailored to the patients
Friedman [50•]	Randomized controlled trial	Community sites (e.g., senior centers) in 29 different communities within the Greater Boston metropolitan area selected to represent demographic diversity of the region	Computer-based telecommunications system developed to monitor and counsel high BP patients on medication adherence and compared to usual care. Patients asked to report: (1) BP; (2) understanding of prescribed medication regimen; (3) adherence; (4) symptoms and side effects	For those in the telephone group (*n* = 133), medication adherence increased (*p* = 0.03) and mean diastolic BP decreased (*p* = 0.02) even without medication adherence (0.01). With medication adherence, the telephone group had a larger decrease in diastolic BP (*p* = 0.03)
*Electronic health record enabled interventions*				
Jaffe [69•]	Quasi-experimental study	An integrated health system in Northern California	Group of randomly selected patients with HTN within the Kaiser Permanente Northern California (KPNC) Hypertension program compared with state and national estimates. HTN control was assessed using EHRs	Patients in the KPNC program had a higher increase in hypertension control (*p* < 0.001) when compared to controls (national and state specific means)
Mulrooney [80•]	Quality improvement	A federally qualified health center with 14 sites throughout Connecticut	Two population health pharmacist (PHP) interventions were evaluated. The PHP completed assessments and sent recommendations using EHRs. The Just-in-time (JIT) approach focused on patients with same week appointments, and the Anytime (ANY) approach focused any patient with uncontrolled HTN regardless of appointment date	JIT (*n* = 37) and ANY (*n* = 41) were assessed using Reach, Effectiveness, Adoption, Implementation, and Maintenance framework dimensions. When implementing a PHP, consider (1) building relationships with medical team; (2) respect providers workload; (3) sent recommendations in a concise, timely, and easy to interpret way; (4) measure effectiveness with defined metrics

Based on the range of system-level factors that impact HTN outcomes and access to care, telehealth and virtual care address system level barriers to care by facilitating improved access. However, system level factors and social determinants of health present unique challenges and opportunities for uptake. For example, there are concerns that telehealth technologies may impact health equity if uptake, particularly for synchronous audio–video technologies, is greater among wealthier, white, and younger patients [72, 73]. Variation of telehealth use is due to multi-factorial drivers including differences in social determinants (e.g., access to a reliable internet connection, patient and provider preferences, technological literacy, condition complexity, and medical visit type) [74–76]. However, evidence from the VA, which serves a significant rural patient population, suggests that telephonic telehealth may be an effective mechanism for improving access to care for more remote, underserved communities [77, 78]. While effective best practices for telehealth continue to emerge across the care continuum, there is a need to advance integration of telehealth approaches within a population health management paradigm (i.e., proactive, focused on a population and equity), requiring complementary team-based models of care and EHR-enabled identification and coordination. To better illustrate the intersection of telehealth and population health management, we present an evidence-based intervention called TEAM [79••].

## Case Study: Team-Supported, EHR-Leveraged, Active Management (TEAM) Intervention

As the largest integrated health care system in the USA, the Veterans Affairs’ Veterans Health Administration (VHA) has a unique opportunity to manage HTN using a population health approach and presents an integrated care environment which allows for a successful intervention because of advancements in care coordination and management [71]. A population health approach in the context of the VHA includes leveraging a team of specialists drawing from the integrated system to work together. Care coordination is an especially critical consideration for rural communities that the VHA disproportionately serves, who face challenges associated with higher rates of HTN and inadequate local resources [81]. To address this challenge and integrate the aforementioned components of effective HTN population health management, we developed Team-supported, EHR-leveraged, Active Management (TEAM) based on stakeholder feedback using existing health care system employees, resources, and technological infrastructure.

**Fig. 1:**
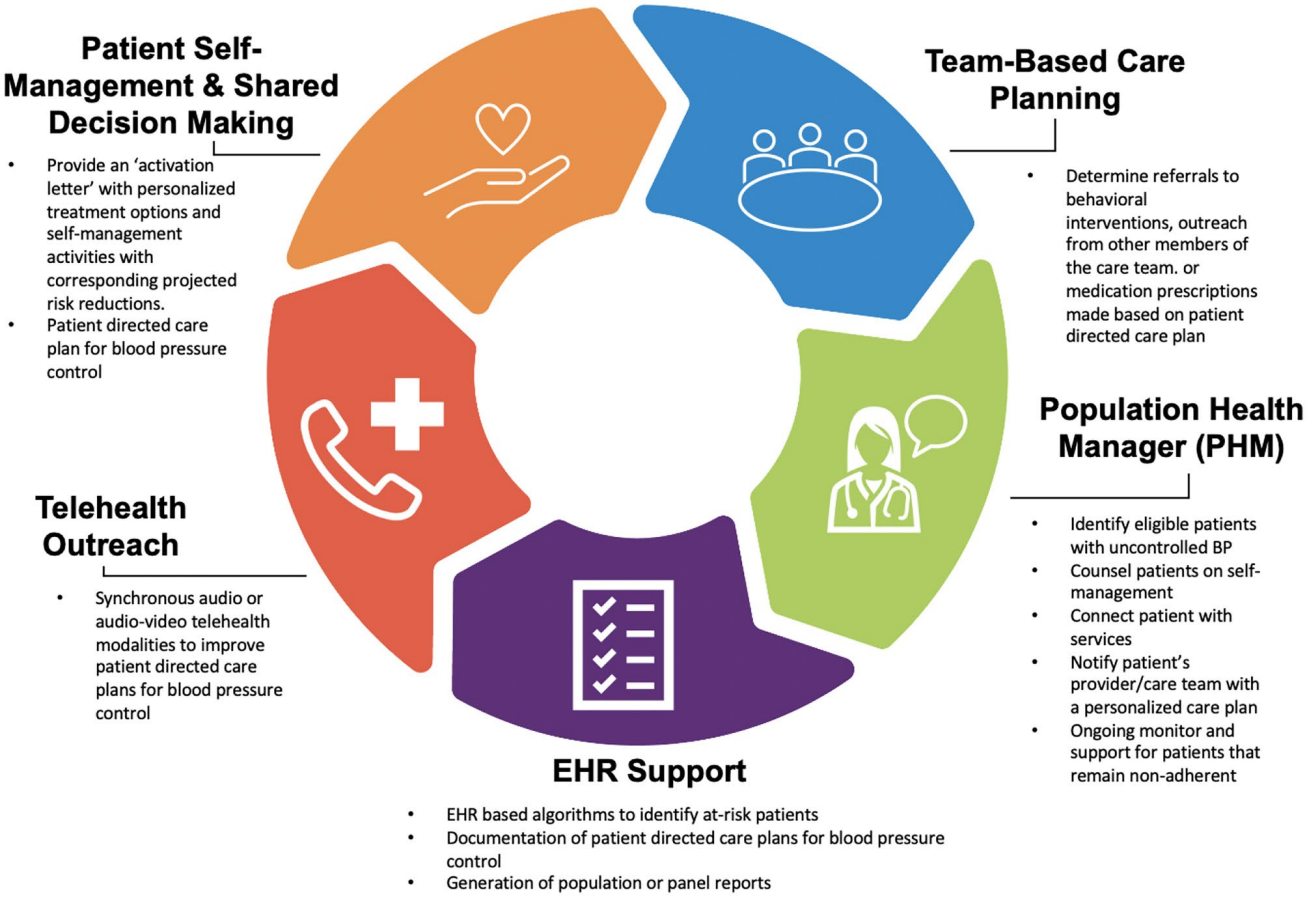
TEAM intervention core components, including team-based care planning, population health manager, EHR support, telehealth outreach, and patient self-management and shared decision-making, represented as an interconnected cycle

TEAM leverages capabilities of an integrated health care system consistent with best practices for improving HTN population health outcomes. TEAM includes a targeted approach to a defined patient population using the electronic health record, a team-based approach featuring a nurse population health manager and leveraging existing telehealth infrastructure. TEAM consists of five core components: (1) identification of patients with uncontrolled HTN for enrollment according to criteria using an EHR-based query; (2) the patients received a CVD risk letter; (3) the population health manager created and entered a detailed care plan based on patient needs; (4) the primary care team ordered appropriate treatment; and (5) the population health manager reported patient progress [79••]. These complementary core components support Veterans in achieving optimal blood pressure control (Fig. 1). The pilot study of TEAM intervention demonstrated improved patient and system level outcomes, including improvements in HTN control [79••]. On the patient level, at 45 days following the TEAM intervention, 40% of patients had blood pressure measurements < 140/90 mm Hg. Compared with baseline (151/95 mm Hg), there was an average reduction of 11 mmHg and 5 mmHg, on systolic and diastolic blood pressure, respectively. This BP improvement was accomplished through improved patient engagement to adopt self-management behaviors and engage in productive interactions with their health care team. After the intervention, 61% of patients scheduled appointments, 83% attended scheduled appointments, and 62% contacted a healthcare provider either check their BP, change medications, or get a referral for a health service [79••]. The promising findings of the TEAM intervention are likely due to the complementary components based on emerging best practices in the HTN population health management literature; however, the integration of TEAM into real-world practices required modifications and adaptations to ensure fit.

### TEAM Adaptations

Few research-developed interventions are adopted into real-world practice settings [82]. When they are, they are regularly modified or adapted to meet the needs of the target population or to match the clinical context. Often this process of modification is driven by constraints or contextual factors (e.g., staffing, cultural considerations, resource availability) and is done without a strategic or purposeful approach to ensure fidelity to the intervention’s original design. To support translational efforts, especially for multi-level interventions, it is critical to carefully and intentionally adapt interventions to match their intended context without compromising the essential elements that make it effective. Herein we describe TEAM intervention adaptation to better integrate in real-world clinical environments within diverse VA clinics. We used the Stirman et al.’s Framework for Reporting Adaptations and Modifications-Expanded (FRAME) to analyze and organize TEAM intervention refinements, additions, and modifications [83, 84]. By doing so, both practitioners and researchers can assess the rationale, timing, relationship to fidelity, and the impact of the modifications to ensure the intervention will retain its effectiveness.

FRAME classifies adaptations across different categories to inform reporting. These include (1) when and how the modification(s) were made, (2) whether it was planned/proactive (i.e., an adaptation) or unplanned/reactive, (3) the stakeholder that determined that the modification should be made, (4) what is modified, (5) the level of delivery that the modification is made (e.g., individual, cohort, practitioner, clinic, network, or system/community), (6) the nature of content-level modifications (e.g., substitution, addition, integrating other evidence based components), (7) the extent to which fidelity is preserved, and (8) the modification rationale (e.g., the intent or goal of the modification or the contextual implementation factors that the modification was made in response to).

We documented the adaptations and changes to the TEAM protocol by engaging with front-line stakeholders, investigators, and research staff. Once feedback had been collated, we organized and reported adaptations to intervention components consistent with FRAME. Adaptations were developed proactively and iteratively at a single site during a pilot phase to identify barriers to implementation and implementation strategies. We focused on adaptations across four core elements of the TEAM intervention: (i) identification and screening of eligible patients; (ii) recruitment and outreach; (iii) patient engagement and activation; (iv): EHR-enabled documentation and team-based care planning. The evaluation of modifications to improve fit with local practice patterns and capabilities can inform the design of implementation strategies and provide practical considerations for practitioners for integrating TEAM and evidence-based HTN population health management approaches like it (see Table 2). For example, we found that adaptations ranged from minor “tweaks” to facilitate adoption (e.g., minor changes to inclusion criteria to reach more patients or utilizing locally available technologies to promote communication and care planning) to more significant alterations of the intervention (e.g., removing medication adjustments as a component of the intervention). These data on implementation and adaptations were used to identify relevant and agreed upon implementation strategies to overcome anticipated implementation barriers as we implemented TEAM at additional subsequent VHA sites. By providing guidance and technical assistance on permissible adaptations that can be made without compromising intervention fidelity, translational efforts to integrate TEAM into real-world clinical settings can be better supported.

**Table 2:** TEAM adaptations, intervention refinements, and modifications organized by the Framework for Reporting Adaptations and Modifications-Expanded (FRAME)

*Original intervention component description*	*Rationale, planning, and stakeholder determination*	*Adaptations and modifications: what, when, how, and level of delivery*	*Nature of modification and extent to which fidelity is preserved*
The TEAM intervention’s **screening and identification processes** operationalized the inclusion/exclusion criteria for receiving the TEAM intervention and consisted of: • > 2 PCP visits within the past year to ensure the VA PCP was managing care rather than an outside PCP • An upcoming visit within the next 2–4 weeks • Uncontrolled CVD risk factors (i.e., blood pressure (BP) and cholesterol), and who were eligible, but not prescribed a statin • Ineligible if patient had no reported cholesterol values within the past two years because a risk profile could not be calculated • Ineligible if patient did not have two primary care visits within the past year Statin prescription but not adherent (e.g., no refill within the last 180 days)	Adaptations of the original approach to TEAM screening and identification processes were made based on the following factors: • PHM reported screening process was too time intensive to fit into clinical workflow • Data pull included past appointment dates and BP values, but did not include upcoming appointments • Missing data impacted screening process to identify eligible Veterans • PHM noted many Veterans were not meeting all clinical and recent visit requirements set forth in the initial eligibility criteria during screening PHM and the study team determined that adaptations should achieve the following goals: • Refine screening process to decrease volume of eligible Veterans and prioritize underrepresented minority and women patients • Optimize PHM’s screening and patient identification effort to ensure it is compatible with clinical workflows • Eligibility criteria were designed to target the intended population of patients that were unengaged • Improve the timeliness of the data for real-time decision making	After initial piloting of the original screening process and challenges were identified, the following changes at the provider level occurred to the screening and population identification process: • Relaxed inclusion criteria to include patients who only had one PCP visit within the last 6 months • Removed requirement that patients must have an upcoming PCP appointment • Removed “AND” criteria to include patients that met some, but not all clinical criteria (e.g., patients who had received a statin refill within the last 180 days, but still had high BP) • List of eligible Veterans only refreshed monthly to streamline identification	The adaptations improved the fit with provider preferences, workflows, data infrastructure and reporting capabilities. By removing or substituting inclusion/exclusion criteria and loosening the structure of the identification/screening process, the PHM changed the target intervention population without compromising the intervention’s potential benefit and expanding potential reach to patients that could benefit
**Recruitment and outreach** of patients in the TEAM intervention is a key consideration for implementation. The process for engaging eligible patients included having the PHM send a *HTN (Hypertension) risk letter*, also referred to as the *Heart Health Handout*, by direct mail or EHR secure message. The letter described HTN risk factors and informed patients of why they had been selected to receive this information. The letter was designed to prepare the patients for a discussion with a member of their health care team and was sent to patients with a scheduled visit, within one week of the scheduled visit. It included personalized treatment options, and information on how self-management activities could impact HTN risk	Adaptations of the original approach to TEAM recruitment and engagement were made based on the following factors: • Veterans were not reading letter and/or keeping primary care appointments. There was a desire to engage even if they were not attending the subsequent medical appointment and, therefore, did not have recent blood pressure values in their medical record • Clinician stakeholders believed the handout did not explain HTN risk well. They did not perceive the handout to be tailored enough or provide flexibility for the unique goals set for the patient • Clinician stakeholders also commented that the proposal risk reduction activities were not realistic • Development of the handout was resource intensive requiring labor to create, populate with personalized content, and mail • Patient stakeholders believed the handout’s graphics were challenging to interpret and did not prioritize what information was critical and needed to be acted upon Clinician and patient stakeholders determined that adaptations should achieve the following goals: • TEAM recruitment and engagement should accommodate patients with frequent missed appointments • The activation letter/*Heart Health Handout* should be more accessible and personalized to patients • Risk reduction and self-management recommendations should be realistic and attainable • Development of the handout should efficiently utilize existing resource to create, populate, and mail	After engaging with clinician and patient stakeholders, the following changes at the patient and provider levels occurred to the recruitment and enrollment process: • Recruitment was no longer connected to an appointment to ensure patients without regular medical appointment attendance would also be included. To do so, patients were selected from a list of patients with a recorded high blood pressure in the last 6 months. Invited patients without upcoming appointments would be called 2 weeks after the handout mailed • The letter/*Heart Health Handout* was adapted in several ways: o Addressed clinician concerns about whether recommendations were attainable or realistic by focusing on blood pressure control as a proxy for overall cardiovascular risk to ensure patients have a better understanding of controlled and/or uncontrolled blood pressure values. Content featured actionable self-management activities the patient could adopt o Emphasis on tailoring the letters based on age, diabetes, and blood pressure control, and corrected the potential improved risk of patients o Changed graphics and imagery to be more accessible to patients and possible to create given printing equipment. Also, numeracy describing HTN risk was simplified, and the order of information and the size of text were modified to indicate priority areas on which to focus o Updated the risk algorithm to describe tailored risk factors so that it was aligned with the clinician’s treatment priority	The adaptations improved the reach, clinical relevance, and accessibility of the intervention for a Veteran patient population. By expanding recruitment to include patients that did not make or keep regular appointments, the TEAM intervention could better engage less activated patients. The handout modifications focused on tailoring information and improving readability in an efficient manner by changing the intervention’s materials and packaging. These changes were done to retain fidelity to the intervention while accommodating clinician and patient stakeholder feedback to improve the fit of TEAM as an element of standard of care
The TEAM intervention leveraged telehealth to **engage and activate** patients based on the tailored *Heart Health Handout*. The PHM’s scope of practice and protocol for engaging patients maintained fidelity to the intervention to convey personalized HTN risks while also being flexible to the individual circumstances and inquiries of the patient. Using existing telephonic encounter infrastructure allowed for efficient contact with patients in-between medical visits	The TEAM intervention components associated with engaging patients via telehealth was adapted based on the follow factors and justifications: • PHM was unable to make medication adjustments because it was outside of their scope of practice and missing blood pressure values in the EHR • Lack of standardized protocol and scripting for engaging the patients during telephonic encounters • Patients did not always receive or read the *Heart Health Handout* • Communication options for contacting eligible patients varied by clinical role • Unclear performance metrics and goals for engaging patients Clinic leadership, clinician, and PHM stakeholders determined that adaptations should achieve the following goals: • PHM role should be re-conceptualized based on their scope of practice • PHM telephonic encounters to engage the patient should be guided by suggested scripting and a protocol framework to support replicability and retain fidelity to the goals of the intervention • Telehealth encounters should be complemented by other existing tools and infrastructure in a coordinated manner • Clear, measurable, time-bound metrics should be established to clarify expectations	Based on the feedback from local stakeholders, the following modifications were made at the patient and provider levels to address the identified factors for engaging patients that inhibited reach and scaling of the TEAM intervention: • PHM role was modified to become more of a ‘health coach’ that entailed motivational discussions and reviewing actionable tasks the patient could take to reduce their blood pressure • Outcome of interest for TEAM shifted from medication changes to goal setting for behavior change • Telehealth encounter scripting and protocols were established to ensure the *Heart Health Handout* was reviewed in detail and prioritized self-management goals consistent with the PHM’s modified scope of practice • Tools and modalities for communicating with patients were defined based on clinical role to ensure coordination o PHM used telephone, e-mail, text, and patient portal messaging o Primary care provider and care team members used exclusively general clinic extension number for escalated issues or clinical concerns • Created a formal and structured metrics and goals process so progress could be documented and tracked	Adaptations to processes for engaging patients improve the potential impact of the TEAM intervention and made it compatible with existing scope of practice policies and locally available technology and infrastructure. The changes to scope of practice resulted in the PHM no longer emphasizing medication adjustments or titration and, instead, focusing on tailored self-management goals to reduce the patient’s blood pressure. These changes were done to improve the fit and compatibility of the intervention within the clinical context. However, since medication changes are no longer part of the intervention, fidelity is inconsistent, and a core element of TEAM has been altered. Additional research is required to determine whether the effectiveness is diminished
**EHR-enabled documentation** was used to ensure the intervention facilitated **team-based care planning.** By using the EHR to document recommendations, encounters, and clinical notes, the PHM could ensure that quick action and communication was supported between members of the patients’ care team	The TEAM intervention’s core components emphasize leveraging the capabilities of the EHR to promote team-based care planning. The following factors motivated adaptations to promote adoption and sustainability by securing buy-in from key provider, and clinic leadership stakeholders: • Low participation and engagement from clinic leadership and providers due to competing priorities and capacity constraints • Operational logistics and planning required established channels of communication between clinical stakeholders • EHR documentation for maintaining patient blood pressure care plan and action steps needed to be shared with relevant members of the care team but PHM did not have EHR permission to attach documentation as an embedded EHR template	The following strategies and adaptations were used to address implementation barriers at the clinic and provider levels: • To address low participation and engagement from providers and leadership, the following actions were taken: o Enhanced credibility and organizational commitment by establishing TEAM intervention as an approved quality improvement project o An in-person meeting with relevant stakeholders to build trust, cooperation, and buy-in o Incentivize participation at the clinic level through making available resources or infrastructure or personnel • Regular, project-specific meetings with front-line stakeholders to monitor progress and address concerns in real time • Utilize auxiliary members of the care team and administrative support to support logistical constraints to upload the blood pressure care plan to the EHR to be available and visible to relevant members of the care team • Developed real-time communication channels between the PHM and primary care provider to ensure timely awareness of the blood pressure plan in the EHR	The adaptations facilitated team-based care by modifying the TEAM intervention to fit local practice patterns related to EHR documentation and care team communication. Since these changes constituted ‘tweaking’ or minor process changes to fit systems and procedures at the clinic level, and did not alter the content, delivery, or intensity of the intervention, it did not impact fidelity. Addressing provider and clinic leadership constraints and lack of engagement likely increased fidelity to the intervention

*PHM* population health manager

### Implementation Considerations and Lessons Learned

Adaptations are a key concept in implementation to improve intervention fit or effectiveness in a specific context. The aforementioned modifications rarely exist within a vacuum and are often made in response to implementation factors. To highlight implementation factors associated with TEAM, we assessed implementation context based on the Consolidated Framework For Implementation Research (CFIR), a framework centered on (1) intervention characteristics, or attributes of TEAM that impact implementation success; (2) outer setting, or economic and social factors within the community and health system that affect implementation; (3) inner setting, or contextual factors within the health system and clinics that influence implementation; (4) individual characteristics of the individuals that use TEAM; and (5) implementation processes, or actions taken by individuals to implement [85, 86]. The experience using a population health manager within a multi-level HTN population health management intervention requires attention to the implementation factors across domains captured by CFIR. In Table 3, we summarize these lessons learned and map them to CFIR domains and constructs to illustrate the complex interplay between the intervention, context, and the dynamic processes, systems, and agents that can drive or inhibit implementation, for example, defining roles and performance metrics and establishing communication channels and protocols for team-based care.

**Table 3:** Key lessons learned and associated Consolidated Framework for Implementation Research (CFIR) domains and constructs

**Associated CFIR domains and constructs**	**Lessons learned**	**Suggestions and implications**
The Inner Setting domain describes the organizational features, characteristics, and culture that influences implementation processes. The *Networks and Communication* nested construct describes the nature and quality of formal and informal communication mechanisms that are specific to an organization	Team-based care planning requires communication through structured channels and mechanisms	• Ensure open and frequent communication between the population health manager and designated members of the care team. This also includes personnel associated with research or quality improvement activities • Leverage EHR enabled features for documentation of intervention to facilitate communication within existing practice patterns
The Characteristics of Individuals domain recognizes that organizations are made up of individuals with beliefs, knowledge, and actions that influences the implementation process. The *Knowledge and Beliefs about the Intervention* nested construct describes how attitudes towards the proposed intervention can shape implementation. Similarly, *Relative Priority* within the Inner Setting domain characterizes individuals’ shared perception of the importance of implementation within the organization	Incorporating key clinical and administrative stakeholder perspectives is critical for implementation success	• Gaining buy-in from key stakeholders during planning will support implementation by preemptively addressing anticipated barriers • Build shared enthusiasm and consensus on the importance of the intervention • Incentivize participation through monetary and non-monetary resources • Understand the “ripple-effect” of the intervention on other clinicians, staff, and administrative support
The Outer Setting domain describes the structural components that influence implementation (e.g., system level policies, laws, and regulations). Specifically, the *External Policies and Resources* nested construct describes the governmental regulations, financing, and reporting that dictate implementation determinants ranging from clinician scope of practice, health service reimbursement, and EHR data sharing	Establishing clear roles, scope of practice, practice patterns, and performance metrics that are consistent with overarching organizational priorities, policies, and procedures	• Understand the limitations to scope of practice for the population health manager and other clinical support roles critical for designing roles within the intervention • Integrate EHR-enabled interventions with attention to the practice patterns for documentation to promote use as an element of standard of care • Link intervention performance metrics to organization-level metrics and goals

Retrieved from https://www.cfirguide.org

## Conclusion

HTN population health management is an important frontier for the field that requires adapting best practices within a population health paradigm consistent with the CCM. We describe relevant literature and discuss the resulting implications for staffing, organization of care, integration of technologies and analytics, and implementation by exploring promising, evidence-based components including leveraging the EHR, team-based care, telehealth, and novel care team composition (e.g., the role of a population health manager). These strategies and components are successful because they advance HTN care in a CCM consistent and organized way within a population health paradigm by being responsive to the multi-level drivers of poor HTN adherence and outcomes.

We use the TEAM intervention as an exemplar that has demonstrated improved patient and system level outcomes, including improvements in HTN control [79••]. Specifically, TEAM improved engagement and management in patients with high BP and led to improved clinical outcomes [79••]. We also explore the challenges to translating intervention components developed in a research context into routine care and the need for strategic and proactive adaptation. In doing so, we highlight the complex interplay and practical considerations of modifying an intervention to facilitate adoption balanced with the importance of maintaining fidelity to evidence-based components. These adaptations range from “tweaks” to substantive modifications and should be monitored carefully to ensure that effectiveness is not diminished. The experience with TEAM in the VA highlights the promise of translational efforts but should be interpreted with caution. As TEAM and interventions like it are moved outside of a VA institutional environment into community and other clinical contexts, further adaptation will be needed in response to dynamic factors including patient characteristics, data availability, and existing infrastructure.

## Data Availability

Upon request from Dr. Bosworth, the project lead of TEAM.
